# Renoprotective Effects of Vortioxetine in Experimental Renal Ischemia–Reperfusion Injury: An Investigation of Oxidative Stress, Inflammation, and Apoptotic Signaling

**DOI:** 10.3390/ijms27198679

**Published:** 2026-09-28

**Authors:** Resul Çiçek, İbrahim Topçu, Bulut Dural, Kevser Tanbek, Evrim Yalçın, Nigar Vardı, Onural Ozhan

**Affiliations:** 1 Department of Urology, Faculty of Medicine, İnönü University, 44280 Malatya, Türkiye; ibrahim.topcu@inonu.edu.tr (İ.T.); bulut.dural@inonu.edu.tr (B.D.); 2 Department of Physiology, Faculty of Medicine, İnönü University, 44280 Malatya, Türkiye; kevser.tanbek@inonu.edu.tr; 3 Department of Histology and Embryology, Faculty of Medicine, İnönü University, 44280 Malatya, Türkiye; evrim.yalcin@inonu.edu.tr (E.Y.); nigar.vardi@inonu.edu.tr (N.V.); 4 Department of Pharmacology, Faculty of Medicine, İnönü University, 44280 Malatya, Türkiye; onural.ozhan@inonu.edu.tr

**Keywords:** acute kidney injury, ischemia–reperfusion injury, vortioxetine, oxidative stress, apoptosis, inflammation

## Abstract

Renal ischemia–reperfusion (I/R) injury is a leading cause of acute kidney injury and is driven by oxidative stress, sterile inflammation, and regulated cell death. Vortioxetine (VOR), a multimodal antidepressant, carries anti-inflammatory and antioxidant activity beyond its neuropsychiatric actions, but has not been tested in renal I/R injury. Thirty-six female Sprague–Dawley rats were allocated to Sham, I/R, or VOR+I/R (10 mg/kg/day by oral gavage) groups. All animals underwent right nephrectomy; in the I/R groups the left renal artery was then clamped for 60 min and reperfused for 23 h. Renal function markers, cytokines, oxidative stress parameters, histopathology, caspase-3 immunoreactivity, and V-cam, Bax, Bcl-2, and p62/SQSTM1 expression were assessed. Ischemia–reperfusion raised serum blood urea nitrogen (BUN), V-cam and Bax expression, and caspase-3 immunoreactivity, and lowered Bcl-2 and p62/SQSTM1; renal interleukin-6 (IL-6), unexpectedly, was lower than in Sham. VOR reduced renal malondialdehyde (MDA) and caspase-3 immunoreactivity, lowered V-cam and Bax and restored Bcl-2 and p62/SQSTM1 in semi-quantitative immunoblots of pooled samples, and limited tubular epithelial desquamation, with Bowman’s space comparable to Sham. BUN and IL-6 in VOR-treated animals were intermediate and did not differ significantly from either Sham or I/R. Serum creatinine, TNF-α, IL-1β, PGC-1α, and most antioxidant parameters were unaffected. VOR thus produced partial, pathway-selective renoprotection rather than uniform suppression of I/R injury. Mortality was numerically higher under VOR, so tolerability at this dose warrants dedicated evaluation.

## 1. Introduction

Acute kidney injury (AKI) remains a common and consequential complication in hospitalized and surgical patients, and renal ischemia–reperfusion (I/R) injury accounts for a substantial share of cases. It arises whenever renal perfusion is interrupted and then restored, as in kidney transplantation, partial nephrectomy, cardiovascular surgery, shock, and sepsis [1]. Loss of blood flow produces hypoxia, ATP depletion, ionic imbalance, and mitochondrial dysfunction; restoring oxygen delivery, although indispensable, imposes a second wave of damage through rapid generation of reactive oxygen species, activation of inflammatory and endothelial responses, and initiation of regulated cell death [1,2,3]. What emerges is therefore not a simple perfusion deficit but a coordinated injury involving tubular, glomerular, vascular, and immune compartments simultaneously.

Oxidative stress (OS) and sterile inflammation are tightly coupled within this process. Excess reactive oxygen species drive lipid peroxidation, protein oxidation, and DNA damage and compromise membrane integrity at a point when endogenous antioxidant defenses are frequently overwhelmed [2,3]. Malondialdehyde (MDA), a downstream product of lipid peroxidation, is widely used to index this component of injury in experimental models. In parallel, damage-associated molecular patterns released from injured tubular cells stimulate cytokine production and endothelial activation. Interleukin-6 (IL-6) and vascular cell adhesion molecule-1 (V-cam) are of particular interest here because they report on inflammatory signaling and on the leukocyte–endothelial interactions that aggravate microvascular dysfunction [1,4]. Apoptosis contributes further to tubular cell loss: the balance between B-cell lymphoma-2-associated X protein (Bax) and B-cell lymphoma-2 (Bcl-2) sets mitochondrial susceptibility, while caspase-3 serves as the principal execution step [5]. Sequestosome-1 (p62/SQSTM1) and peroxisome proliferator-activated receptor gamma coactivator-1 alpha (PGC-1α) offer complementary information on autophagy-related stress responses and mitochondrial adaptation [4,6].

Vortioxetine (VOR) is a multimodal antidepressant that inhibits the serotonin transporter while modulating several serotonergic receptors [7,8]. Interest in the drug has broadened beyond psychiatry: in human monocyte and macrophage models it suppresses oxidative activity, dampens pro-inflammatory pathways, and shifts macrophage polarization toward an anti-inflammatory phenotype [9]. Comparable protection has since been reported in cerebral ischemia and in several models of chemotherapy-induced tissue injury. Evidence in the kidney is confined to a single report in which VOR attenuated gentamicin-induced renotoxicity in rats [10]. Whether VOR is protective in renal I/R injury—in which reperfusion-driven oxidative stress, endothelial activation, and regulated cell death predominate—has not been examined, and the question is worth asking given how closely inflammation, OS, and apoptosis are intertwined in this setting.

We therefore tested whether VOR limits renal injury in a uninephrectomized rat model of left renal ischemia–reperfusion. Renal function, oxidative and inflammatory markers, endothelial activation, apoptosis-related proteins, p62/SQSTM1, PGC-1α, caspase-3 immunoreactivity, and histopathological change were assessed. Our hypothesis was that VOR would attenuate injury by reducing lipid peroxidation, inflammatory and endothelial activation, and apoptotic signaling.

## 2. Results

### 2.1. Experimental Flow, Mortality, and Weight Parameters

Several animals died during the experimental period, so analyses were restricted to those completing the protocol. Of the 12 animals allocated to each group, 11 in the Sham group, 11 in the I/R group, and 9 in the VOR+I/R group were analyzed (Table 1). Deaths occurred in one Sham animal (Rat No. 4), one I/R animal (Rat No. 10), and three VOR+I/R animals (Rat Nos. 9, 11, and 12). All deaths occurred after right nephrectomy and renal ischemia (or the corresponding procedural stage in the Sham group), that is, during the post-operative reperfusion period; none occurred during the pretreatment period. Mortality did not differ significantly across the three groups (Fisher–Freeman–Halton exact test, *p* = 0.584).

Baseline body weight, final body weight, and kidney weight were comparable across groups (Table 2). Body weight change over the experimental period did differ (one-way ANOVA, *p* = 0.002): VOR+I/R animals lost more weight (−10.9 ± 8.1 g; −4.96 ± 3.47%) than I/R animals (−1.4 ± 3.3 g; −0.59 ± 1.47%; Tukey *p* = 0.001), with the Sham group intermediate (−5.9 ± 3.6 g; −2.65 ± 1.63%) and not significantly different from either (*p* > 0.05). Relative kidney weight was similar across groups (Sham 4.04 ± 0.34, I/R 3.81 ± 0.41, VOR+I/R 3.93 ± 0.55 mg/g; ANOVA *p* = 0.471).

### 2.2. Renal Function and Inflammatory Markers

Serum BUN rose significantly in the I/R group relative to Sham (*p* < 0.05), whereas BUN in the VOR+I/R group was intermediate and did not differ significantly from either I/R or Sham (*p* > 0.05). Serum CR, by contrast, did not differ across groups (*p* > 0.05).

Among the inflammatory markers, renal IL-6 was significantly lower in the I/R group than in Sham (*p* < 0.05); in the VOR+I/R group IL-6 was intermediate and did not differ significantly from either Sham or I/R (*p* > 0.05). Neither IL-1β nor TNF-α differed between groups, and renal PGC-1α was likewise unchanged (*p* > 0.05). Results are shown in Figure 1. Comparisons in this section used one-way ANOVA followed by Tukey’s multiple-comparisons test.

### 2.3. Oxidative Stress Parameters

Renal MDA, an index of lipid peroxidation, was higher in the I/R group than in Sham but not statistically significantly (*p* > 0.05). VOR reduced MDA relative to untreated I/R (*p* < 0.05), to a level that was also significantly below Sham (*p* < 0.05).

The antioxidant defense parameters (Figure 2) SOD, CAT, GSH, GSH-Px, and TAS showed no significant between-group differences, and neither did PC, TOS, or OSI (*p* > 0.05). 

### 2.4. Western Blot Findings

V-cam expression differed across groups. It was higher in the I/R group than in Sham (*p* < 0.05), and although VOR reduced it relative to untreated I/R (*p* < 0.05), expression remained above Sham levels (*p* < 0.05).

Bax expression was increased in the I/R group compared with Sham (*p* < 0.05). VOR reduced Bax relative to untreated I/R (*p* < 0.05), to a level not different from Sham (*p* > 0.05).

Bcl-2 followed the opposite pattern: expression fell in the I/R group relative to Sham (*p* < 0.05) and was restored by VOR (*p* < 0.05).

p62/SQSTM1 expression was lower in the I/R group than in Sham (*p* < 0.05) and was restored by VOR (*p* < 0.05), with no difference between VOR+I/R and Sham (*p* > 0.05). Results are shown in Figure 3. Because each group was represented by a single pooled homogenate blotted in five technical replicates, these comparisons reflect differences between pooled samples and assay reproducibility rather than inter-animal variability; the Western blot data are therefore semi-quantitative and supportive, and the associated *p* values are reported descriptively. Comparisons in this section used one-way ANOVA followed by Tukey’s multiple-comparisons test.

### 2.5. Histopathological Findings

In Sham animals the architecture of renal cortex and medulla was largely intact, with only mild tubular epithelial desquamation and minimal glomerular and medullary congestion and no prominent degenerative change (Figure 4).

The I/R group showed marked structural disruption: widespread tubular epithelial desquamation, hydropic degeneration, cellular debris within tubular lumina, and glomerular congestion. Bowman’s space was visibly widened and glomerular capillaries engorged with erythrocytes (Figure 4). Histomorphometry confirmed that Bowman’s space was wider in the I/R group than in Sham (15.60 [11.20–20.50] µm versus 11.90 [9.20–15.30] µm; *p* < 0.05). Glomerular congestion (1.37 [0.85–1.75] versus 0.47 [0.35–0.50]), tubular desquamation (1.85 [1.20–2.60] versus 0.65 [0.20–0.70]), and hydropic degeneration (1.70 [1.10–2.60] versus 0.10 [0.00–0.20]) scores were all significantly increased (*p* < 0.05).

The VOR+I/R group retained tubular epithelial integrity to a greater degree, with fewer desquamated cells in tubular lumina and Bowman’s space measurements comparable to Sham (Figure 4). Bowman’s space width was 11.50 (9.30–13.80) µm and did not differ from Sham (*p* > 0.05), although it was not significantly narrower than in the I/R group either. Tubular desquamation score was lower than in the I/R group (1.20 [0.80–2.00]; *p* < 0.05). Glomerular congestion (0.95 [0.35–2.00]) and hydropic degeneration (1.35 [1.20–1.90]) scores were also lower, but not significantly so (*p* > 0.05).

Glomerular tuft diameter was similar across groups (Sham 83.00 [73.10–88.80] µm; I/R 83.90 [79.80–87.50] µm; VOR+I/R 86.30 [80.40–95.00] µm; *p* > 0.05), as were medullary congestion scores (*p* > 0.05). Comparisons in this section used the Kruskal–Wallis test followed by Dunn’s multiple-comparisons test. Quantitative data are summarized in Table 3.

### 2.6. Immunohistochemical Findings

Caspase-3 immunostaining was weak and confined to tubular epithelial cells in the Sham group. The I/R group, in contrast, showed marked and widespread cytoplasmic brown staining consistent with a substantial rise in caspase-3 activation (Figure 5).

Semiquantitative H-score analysis confirmed higher caspase-3 immunoreactivity in the I/R group than in Sham (5.65 [3.90–8.80] versus 1.45 [0.50–2.40]; *p* < 0.05).

VOR treatment markedly reduced caspase-3 immunoreactivity relative to untreated I/R, with a median H-score of 2.95 (1.50–4.80) (*p* < 0.05). Comparisons in this section used the Kruskal–Wallis test followed by Dunn’s multiple-comparisons test; values also appear in Table 3.

## 3. Discussion

This study examined the effects of VOR on renal function, inflammatory responses, OS, apoptosis, autophagy-related protein expression, and histopathology in an experimental model of renal I/R injury. Ischemia–reperfusion raised BUN, V-cam and Bax expression and caspase-3 immunoreactivity, lowered Bcl-2 and p62/SQSTM1, and produced marked tubular and glomerular damage. VOR treatment lowered MDA, V-cam, Bax, and caspase-3, restored Bcl-2 and p62/SQSTM1, and reduced tubular epithelial injury, whereas its effects on BUN and IL-6 did not reach significance. Taken together, these findings indicate renoprotection at biochemical, molecular, and histopathological levels—though, as set out below, protection was selective rather than global.

Renal I/R injury is among the most extensively studied experimental models of AKI and is characterized by hypoxia, reperfusion-driven OS, endothelial activation, inflammatory cell infiltration, and tubular epithelial death [1,2]. Bonventre and Yang emphasized that ischemic AKI extends well beyond tubular epithelial injury to encompass endothelial dysfunction, inflammatory cell recruitment, and microvascular damage [1]. Recent systematic reviews identify renal I/R injury as a persistent clinical problem, particularly in kidney transplantation, and underline the need for effective pharmacological intervention [11].

The elevation of serum BUN after I/R indicates that the model produced genuine functional injury. BUN in VOR-treated animals was intermediate between Sham and I/R and did not differ significantly from either, so a functional benefit of VOR could not be demonstrated in this model. Serum CR, however, was unchanged. Several factors plausibly contribute: the relatively short reperfusion interval, the modest sample size, and assessment at a single post-reperfusion time point. Previous experimental work has similarly shown that BUN and CR responses after renal I/R depend heavily on ischemia duration, reperfusion time, nephrectomy protocol, and animal age [1,12,13]. Because all groups were uninephrectomized, the Sham group represents a solitary-kidney control rather than an intact control. This design isolates the effect of ischemia–reperfusion from that of reduced nephron mass, but it also means that baseline renal function in every group already reflects a 50% reduction in functional nephron mass, which may compress the dynamic range of serum creatinine and contribute to the absence of a significant between-group difference in this analyte. During peer review, a stoichiometric inconsistency was identified between the originally reported serum urea and BUN values obtained from the same samples: because urea and BUN reflect the same underlying nitrogenous analyte and are therefore not independent measures, a divergence of this magnitude could not be attributed to normal biological or assay variability. As this discrepancy could not be resolved against the original assay documentation, we removed the unvalidated serum urea data from the manuscript and report the validated BUN measurements alone as the index of azotemia in this study.

Contrary to our hypothesis, renal IL-6 was lower in the I/R group than in Sham, and IL-6 in VOR-treated animals was intermediate and did not differ significantly from either group. IL-6 is a well-established mediator of the inflammatory response after renal ischemia and is closely tied to tissue damage, endothelial activation, and leukocyte infiltration [1,4]. Its tissue concentration, however, changes rapidly during reperfusion, and a single measurement after 23 h of reperfusion may have captured a phase in which local IL-6 had already declined. Because all animals underwent right nephrectomy, the surgical insult itself may also have raised IL-6 in the Sham group and narrowed the contrast with I/R. Talmon et al. showed that VOR suppresses oxidative responses and TNF-α production in human monocyte/macrophage cultures, blocks NF-κB nuclear translocation, and promotes anti-inflammatory macrophage polarization [9], and more recent work has reported reductions in inflammation, OS, and apoptosis across several models of tissue injury [14,15]. Our cytokine data do not confirm this anti-inflammatory profile in renal I/R injury: IL-6 did not behave as expected, and IL-1β and TNF-α did not differ between groups, possibly reflecting the time-dependence of cytokine responses, as these mediators may already have peaked or not yet risen at the time of tissue collection. The anti-inflammatory component of VOR’s action in this model should therefore be regarded as unproven, and the reduction in V-cam described below is the more robust inflammatory-endothelial finding. 

PGC-1α likewise showed no between-group differences. As a central regulator of mitochondrial biogenesis, oxidative metabolism, and cellular energy homeostasis, PGC-1α has been implicated in maintaining mitochondrial integrity after renal ischemia, and reduced expression is thought to contribute to mitochondrial dysfunction and impaired recovery. Its stability here may reflect the short reperfusion period or limited sample size, or may indicate that VOR acts predominantly through anti-inflammatory and anti-apoptotic routes rather than by directly modulating mitochondrial biogenesis. Longer reperfusion intervals and mechanistic approaches will be needed to resolve this [16].

Oxidative stress is central to renal I/R injury. Restoration of oxygen supply during reperfusion drives excessive ROS production via mitochondrial dysfunction and the electron transport chain, producing lipid peroxidation, protein oxidation, DNA damage, and loss of membrane integrity [2,3]. The modest, non-significant rise in MDA after I/R suggests that lipid peroxidation in this model may have been partly transient and already subsiding by 23 h of reperfusion. That VOR nonetheless lowered MDA below Sham levels implies that the drug may restrain lipid peroxidation beyond simply offsetting the ischemic insult, possibly by reducing the baseline oxidative load imposed by anesthesia and nephrectomy; without a VOR-only group, however, an effect specific to I/R-induced peroxidation cannot be distinguished from a more general antioxidant action. Comparable antioxidant activity has been reported previously: Caruso et al. found that VOR alleviated OS-related change and modulated antioxidant gene expression in an Alzheimer’s disease model [17], and Emam et al. demonstrated neuroprotection in cerebral I/R through suppression of OS, apoptosis, inflammation, and endoplasmic reticulum stress signaling [18]. In the kidney specifically, 5-methoxytryptophan has been shown to alleviate renal I/R injury by reducing OS and inflammation while suppressing endoplasmic reticulum stress-mediated apoptosis via Nrf2/HO-1 activation [19].

The MDA result stands, however, against a largely negative oxidative panel: SOD, CAT, GSH, GSH-Px, TAS, TOS, OSI, and protein carbonyl were all unchanged. Rather than a global antioxidant effect, then, VOR appears to act narrowly on lipid peroxidation. Two considerations temper this interpretation. OS biomarkers change dynamically over the course of renal I/R injury [2,3], so assessment at a single time point may miss transient shifts in antioxidant defenses. Global indices such as TAS and TOS also carry greater biological variability than specific markers of oxidative damage. Even so, the discrepancy is substantial, and the antioxidant component of VOR’s action in this model should be regarded as unproven pending a time-course study. A further constraint is analytical: SOD, CAT and GSH-Px were measured as protein concentrations rather than as enzymatic activities, and antioxidant capacity depends on catalytic activity rather than on enzyme abundance alone [20]. The unchanged values for these parameters therefore cannot be read as definitive evidence that antioxidant defenses were preserved.

Western blotting revealed a marked increase in V-cam after I/R, reinforcing the role of endothelial activation and inflammatory cell adhesion in this injury. V-cam is upregulated in endothelial and renal cells in response to pro-inflammatory cytokines, facilitating leukocyte adhesion, transmigration, and amplification of inflammation [21,22]. Akhtar et al. reported that V-cam expression rises after renal I/R injury and can be detected by non-invasive molecular imaging [21], and Burne et al. established the importance of adhesion molecules in post-ischemic inflammatory responses [22]; more recent experimental work confirms that upregulation of both ICAM-1 and V-cam is characteristic of renal I/R injury [23]. In our pooled-sample immunoblots VOR reduced V-cam relative to untreated I/R, suggesting its protective effects extend past cytokine modulation to endothelial inflammatory activation. Expression nonetheless remained above Sham levels, indicating partial rather than complete suppression.

Apoptosis is a principal route of tubular epithelial cell loss during renal I/R injury [5,24]. Within the mitochondrial pathway, increased Bax alongside decreased Bcl-2 promotes mitochondrial outer membrane permeabilization and downstream caspase activation [5,24]. Wei et al. demonstrated that Bax and Bak are essential to ischemic AKI, particularly in mediating proximal tubular epithelial apoptosis [24]. Our data reproduce this pattern—elevated Bax and reduced Bcl-2 after I/R—and, in pooled-sample immunoblots, VOR both reduced Bax and restored Bcl-2 toward Sham levels. The anti-apoptotic effect thus appears to involve both suppression of pro-apoptotic signaling and reinforcement of survival signaling, in keeping with earlier reports on VOR and apoptosis-related markers in other tissue injury models [14,15].

Immunohistochemistry corroborated the Western blot findings. Caspase-3 immunoreactivity rose sharply after I/R and was significantly attenuated by VOR. As the principal executioner caspase, caspase-3 is a well-established marker of apoptotic death and has been implicated in both tubular epithelial injury and microvascular dysfunction after renal I/R [5,6,25]. Yang et al. identified caspase-3 as a key regulator of post-ischemic microvascular rarefaction and renal fibrosis [25], and Lan et al. showed that caspase-3-dependent peritubular capillary dysfunction may drive the transition from AKI to chronic kidney disease [6]. The lower caspase-3 H-score in VOR-treated animals, together with the more favorable Bax/Bcl-2 balance, indicates attenuation of mitochondrial apoptotic signaling. Because the Western blot data derive from pooled homogenates and caspase-3 was scored semiquantitatively, these results are best read as anti-apoptotic modulation rather than as proof of blockade of the apoptotic pathway.

Autophagy plays a dual role in renal I/R injury. Basal, tightly regulated autophagy supports cellular homeostasis by clearing damaged organelles and protein aggregates, whereas impaired or insufficient autophagic flux aggravates cellular stress and tissue injury [26,27,28]. Kaushal and Shah emphasized the protective role of autophagy in AKI, particularly in preserving proximal tubular epithelial integrity [26], and Liu et al. showed that autophagy is required to maintain tubular cell homeostasis and protect against aging- and I/R-induced injury [28]. Recent work suggests that assessing LC3-II/LC3-I, Beclin-1, Rab11a, p62, and caspase-3 together provides a more complete picture of the autophagic response [29]. In our pooled-sample immunoblots p62 was lower after I/R and returned toward Sham levels with VOR, but this finding must be interpreted cautiously, both because the Western blot analysis was based on pooled homogenates and on biological grounds. p62 reflects not only autophagy activation but also autophagic degradation, proteotoxic stress, and adaptive cellular responses [26,27,28,29], so its expression alone cannot define autophagic activity. VOR may influence autophagy-related stress responses, but no firm conclusion is possible without LC3-II/LC3-I conversion, Beclin-1 expression, and flux measurements.

Histopathology tracked the biochemical and molecular results. Renal I/R injury widened Bowman’s space and increased glomerular congestion, tubular epithelial desquamation, and hydropic degeneration, indicating structural damage across both tubular epithelium and glomerular microvasculature—consistent with the established pathophysiology, in which tubular epithelial cells are especially vulnerable to hypoxia and reperfusion-driven OS [1,2]. Under VOR, tubular epithelial desquamation was significantly lower than in untreated I/R animals, and Bowman’s space no longer differed from Sham, although the reduction relative to the I/R group did not itself reach significance. Glomerular congestion, hydropic degeneration, and medullary congestion scores were also lower under VOR but did not reach significance. Histological benefit therefore appears unevenly distributed across renal compartments, concentrated in the tubular epithelium where OS and apoptosis contribute most.

VOR was administered by oral gavage to ensure delivery of the complete body-weight-adjusted daily dose and to avoid variability resulting from changes in food or water intake during the perioperative period. This route was also selected for translational relevance because VOR is administered orally in clinical practice and has an absolute oral bioavailability of approximately 75% [30]. Nevertheless, gavage is a laboratory dosing technique that does not fully reproduce voluntary drug intake and may introduce restraint-related stress, although the vehicle-treated groups underwent the same procedure. Alternative enteral approaches in rodents include voluntary administration in a palatable vehicle or incorporation into chow or drinking water. Although these methods more closely resemble spontaneous human administration, the actual dose consumed may be less certain, particularly when surgery or treatment alters appetite. In humans, the corresponding clinically applicable formulations are oral tablets and, where available, oral drops. Parenteral administration would allow controlled experimental exposure but has lower translational relevance because VOR has no approved injectable formulation.

At present, VOR should not be considered an established nephroprotective treatment. Its approved clinical indication remains major depressive disorder, and no clinical study has yet demonstrated that VOR prevents or treats ischemic AKI. If protection were confirmed at clinically relevant exposures, its most plausible application would be short-term perioperative prophylaxis in settings where renal ischemia can be anticipated, such as kidney transplantation, partial nephrectomy, or major cardiac or aortic surgery, rather than prolonged treatment solely for renal protection. Such use would nevertheless represent drug repurposing and would require dedicated dose-ranging, pharmacokinetic, safety, and efficacy trials.

The present five-day pretreatment regimen and 23-h follow-up cannot establish the durability of protection or the consequences of prolonged administration. VOR has a terminal half-life of approximately 66 h and generally reaches steady-state concentrations within two weeks; therefore, the regimen used here did not represent steady-state exposure [30]. Although clinical studies at approved doses of 5–20 mg/day suggest that VOR is generally tolerated during treatment lasting up to 52 weeks [31], these data relate to patients with major depressive disorder and do not demonstrate long-term renal benefit or safety at the higher experimental exposure used here. Prolonged or perioperative use would require consideration of nausea and other gastrointestinal effects, hyponatremia, increased bleeding risk in patients receiving antiplatelet or anticoagulant therapy, serotonergic and CYP-mediated drug interactions, sexual dysfunction, and discontinuation symptoms [31]. These concerns are particularly relevant to surgical and critically ill patients and, together with the mortality and weight-loss signals observed in the present study, preclude direct clinical extrapolation.

Among other serotonergic antidepressants, fluoxetine has attenuated functional, inflammatory, and oxidative alterations in experimental renal I/R injury, while fluvoxamine has improved renal function and survival through sigma-1 receptor-dependent signaling [32,33,34]. However, these findings remain preclinical. Neither these drugs nor other antidepressants are currently approved or recommended as nephroprotective therapy, and their experimental effects should not be interpreted as evidence of a clinically established antidepressant class effect.

A notable safety signal deserves emphasis rather than relegation to a limitations list. Mortality was numerically higher in the VOR+I/R group (25.0%) than in the Sham and I/R groups (8.3% each), and VOR-treated animals lost the most body weight over the experimental period (ANOVA *p* = 0.002). The between-group mortality difference did not reach statistical significance (Fisher–Freeman–Halton exact test, *p* = 0.584), but the study was not powered to detect a difference of this magnitude, and survivor bias cannot be excluded: because all deaths occurred during the post-operative reperfusion period (see below), the most severely affected VOR-treated animals may not have completed the protocol, which would inflate the apparent benefit in those that did. Interspecies scaling places this exposure well above the clinical range: body-surface-area normalization (rat-to-human conversion factor 6/37) gives a human equivalent dose of approximately 1.6 mg/kg, or roughly 100 mg daily for a 60 kg adult, which is five- to ten-fold the licensed dose of VOR (10–20 mg/day). The dose used here was selected for consistency with earlier rodent studies rather than for clinical comparability, and the mortality and weight-loss signals should be read in that light. Any claim of renoprotection at 10 mg/kg/day must therefore be paired with dedicated tolerability evaluation, including dose-ranging and a VOR-only arm without ischemia.

A further strength is the model itself: the uninephrectomized design mirrors the clinical settings in which renal I/R injury is most consequential, namely partial nephrectomy, living-donor nephrectomy, and kidney transplantation. Another strength is the breadth of the work: rather than resting on a single biomarker, it evaluates functional, biochemical, oxidative, inflammatory, apoptotic, autophagy-related, and histopathological endpoints together. The protective pattern across MDA, V-cam, Bax, Bcl-2, p62/SQSTM1, caspase-3, and tubular histology supports the idea that VOR modulates several pathophysiological mechanisms simultaneously—a pattern in keeping with its multimodal pharmacology [7,8]. Mørk et al. showed that VOR acts through multiple serotonergic receptors in addition to serotonin transporter inhibition [7], and Sanchez et al. detailed the properties distinguishing it from conventional SSRIs [8]. Together with previously reported anti-inflammatory, antioxidant, and anti-apoptotic activity across experimental models [9,14,15,17,18], this provides a coherent rationale for pursuing VOR in renal I/R injury.

Importantly, all deaths occurred after nephrectomy and renal ischemia (or, in the Sham group, after the corresponding procedural stage), that is, during the post-operative reperfusion period, and none occurred during the five-day VOR pretreatment period. The occurrence of deaths in both the Sham and untreated I/R groups indicates a background contribution from anesthesia, nephrectomy, surgical stress, or acute postoperative instability. However, because mortality was numerically higher in the VOR+I/R group and no diagnostic necropsies were performed, neither VOR-related toxicity nor an interaction between the high VOR exposure and the surgical insult can be excluded. Future studies should incorporate a VOR-only group, clinically closer dose levels, systematic physiological and behavioral monitoring, plasma VOR measurements, and diagnostic necropsy of animals that die before the planned endpoint.

Several limitations qualify these conclusions. Only one dose was tested, so no dose–response relationship could be established. The study was confined to early reperfusion; renal recovery, fibrosis, and chronic structural change were not examined. Autophagy assessment rested on p62 alone, without LC3-II/LC3-I, Beclin-1, or ultrastructural evaluation of autophagosomes. Mechanistic validation using pharmacological inhibitors or genetic manipulation lay outside the scope of the work. The design lacked a VOR-only group, so drug effects independent of ischemia cannot be excluded. Only female rats were used, and sex-specific responses were therefore not addressed. Antioxidant enzymes were quantified as protein concentrations rather than as catalytic activities, and renal function analytes were measured by immunoassay rather than by conventional enzymatic or kinetic methods, which limits comparability with studies using standard clinical-chemistry platforms. Western blot data reflect five technical replicates from a single pooled homogenate per group rather than five independently blotted animals, so they should be read as a semi-quantitative, confirmatory analysis that does not capture inter-animal biological variability; the associated *p* values describe differences between pooled samples and should not be interpreted as evidence of inter-animal statistical significance. Finally, the absence of change in serum CR, TNF-α, IL-1β, and most antioxidant parameters, the non-significant effect on BUN, and the unexpected IL-6 response indicate that protection does not extend uniformly across the pathways engaged by renal I/R injury.

## 4. Methods and Materials

### 4.1. Animals and Study Design

The study was carried out at the Experimental Animal Production and Research Center of İnönü University, Malatya, Türkiye, using 36 female Sprague–Dawley rats aged 10 weeks and weighing 195–249 g. Animals were housed at 22 ± 2 °C on a 12 h light/12 h dark cycle with free access to standard chow and water. Female rats were used to keep sex constant rather than to vary it: they show less inter-individual variation in body weight and less fighting-related injury when group-housed, and the renal I/R protocol adopted here was established in female animals [35].

The protocol received approval from the Local Animal Ethics Committee of İnönü University (Approval No. 2025/13-9; 17 September 2025) and was conducted according to institutional and national guidelines for laboratory animal care. Reporting follows the ARRIVE 2.0 guidelines [36]; the completed ARRIVE 2.0 checklist is provided as File S1.

Group size was set by a priori power analysis for a three-group one-way analysis of variance (ANOVA). Assuming α = 0.05, β = 0.20 (power = 80%), and a large standardized effect size (Cohen’s *f* = 0.6), 10 animals per group (total *N* = 30) were required. Because peri-operative and post-ischemic mortality is intrinsic to this model, and to preserve power across the full panel of Western blot, ELISA, oxidative stress, immunohistochemical, and histopathological analyses, 12 animals were allocated per group (total *N* = 36). Sample size was determined in line with the principles of replacement, reduction, and refinement (3Rs).

Animals were assigned to groups by a random allocation rule generated by software developed at the Department of Biostatistics and Medical Informatics, İnönü University. Because the sham procedure differed from the I/R procedure only in the application of the vascular clamp, the surgeon could not be blinded to the sham versus I/R condition; allocation to vehicle or VOR was, however, concealed from the surgeon. All subsequent biochemical, molecular, histopathological, and immunohistochemical assessments were performed by investigators blinded to group identity. Only the group coordinator held the allocation code, which was broken after data analysis was complete. All animals in the three groups underwent midline laparotomy and right nephrectomy under identical anesthesia. The groups differed only in whether the left renal artery was occluded and in the substance received by oral gavage. The three groups were:

- Sham: Midline laparotomy with right nephrectomy and exposure of the left renal pedicle without occlusion, preceded by 2 mL distilled water by oral gavage once daily for 5 days.
- I/R: Right nephrectomy followed by 60 min left renal artery clamping and 23 h reperfusion, preceded by 2 mL distilled water by oral gavage once daily for 5 days.
- VOR+I/R: The same I/R protocol, with VOR 10 mg/kg/day in a total volume of 2 mL by oral gavage once daily for 5 days before ischemia.

### 4.2. Renal Ischemia–Reperfusion Model

Anesthesia was induced with intraperitoneal ketamine hydrochloride (75 mg/kg) and xylazine hydrochloride (10 mg/kg). After midline laparotomy, the right kidney was removed and the left renal artery occluded with an atraumatic microvascular clamp for 60 min. The clamp was then released and reperfusion allowed to proceed for 23 h, so that the total interval from clamp application to tissue collection was 24 h, matching the reperfusion window most commonly used in rat renal I/R models [13,35]. Sham animals underwent the same anesthesia, laparotomy, and right nephrectomy; the left renal pedicle was exposed but not occluded, and the abdomen was closed after an equivalent operative interval, so that anesthesia duration, surgical trauma, and nephron mass were matched across groups. In all groups the abdomen was closed in layers and the animals were allowed to recover from anesthesia, with free access to food and water until the end of the observation period. Postoperative analgesia was provided with a single intraperitoneal dose of paracetamol 100 mg/kg. At the end of the corresponding period animals were deeply anesthetized with ketamine (100 mg/kg) and xylazine (10 mg/kg) intraperitoneally and euthanized by surgical exsanguination, during which blood samples were collected. The left kidney was excised, decapsulated, and divided longitudinally; one portion was fixed in 10% formalin for histopathology and immunohistochemistry, and the remainder stored at −80 °C for biochemical and molecular work [35].

### 4.3. Preparation and Administration of Vortioxetine

VOR was suspended fresh in distilled water and given by oral gavage at 10 mg/kg/day in a total volume of 2 mL [18,37]. The dose follows earlier experimental work demonstrating neuroprotection by VOR, including attenuation of OS-related injury in cerebral I/R models [18]. The same 10 mg/kg/day dose and 5-day pre-treatment schedule have also been applied in a rat model of gentamicin-induced renotoxicity [10]. Commercially available Fonksera^®^ 10 mg film-coated tablets (Lundbeck İlaç Ticaret Ltd. Şti., Istanbul, Türkiye) were used as the source of VOR. Each tablet contained 12.71 mg VOR hydrobromide, equivalent to 10 mg VOR. The listed excipients were mannitol (E421), microcrystalline cellulose, hydroxypropyl cellulose, sodium starch glycolate type A, magnesium stearate, and a film coating consisting of hypromellose, macrogol 400, titanium dioxide (E171), and yellow iron oxide (E172). The tablets were finely powdered and freshly dispersed in distilled water before administration. The dose was calculated according to the labeled VOR content and adjusted to each animal’s body weight to provide 10 mg/kg/day by oral gavage in a total volume of 2 mL. Gavage was performed once daily on five consecutive days before surgery (five doses in total), with the last dose given on the day before surgery. No dose was given on the day of surgery or during the 23 h reperfusion period. Sham and I/R animals received an equivalent volume of distilled water by gavage. 

### 4.4. Biochemical Analyses

Blood collected at the end of the protocol was centrifuged at 2500 rpm for 7 min to obtain serum. Creatinine (CR) (Cat No:YLA0560RA) and blood urea nitrogen (BUN) (Cat No:YLA0103RA) were measured as indices of renal function using rat-specific ELISA kits (YL-Biont, Shanghai YL Biotech Co., Ltd., Shanghai, China) according to the manufacturer’s instructions. Serum urea was also initially measured (Cat No:YLA0990RA); however, on re-examination during peer review these values could not be reconciled on a stoichiometric basis with the paired blood urea nitrogen (BUN) measurements obtained from the same samples, and the urea data were therefore excluded from the final analysis and are not reported.

Renal tissues obtained after sacrifice were frozen and stored at −80 °C for biochemical analyses. On the day of analysis, tissues were homogenized in ice-cold potassium phosphate buffer at a ratio of 1:10 (*w*/*v*) (pH 7.4) supplemented with a protease inhibitor cocktail (Abcam, ab201111, Cambridge, UK). Homogenates were centrifuged at 10,000× *g* for 5 min at 4 °C, and the resulting supernatants were used for ELISA analyses. Protein for Western blotting was extracted separately in RIPA buffer, as described in Section 4.5. Commercial ELISA kits (YL biont) were used according to the manufacturer’s instructions. Results were normalized to the total protein content of the homogenates. Interleukin-1β (IL-1β; Cat. No. YLA0153RA), IL-6 (Cat. No. YLA0031RA), tumor necrosis factor-α (TNF-α; Cat. No. YLA0118RA), and PGC-1α (Cat. No. YLA0039RA) were quantified with rat-specific ELISA kits (YL-Biont, Shanghai YL Biotech Co., Ltd., Shanghai, China).

Oxidative status was characterized by measuring renal MDA, superoxide dismutase (SOD), catalase (CAT), glutathione (GSH), glutathione peroxidase (GSH-Px), protein carbonyl (PC), total antioxidant status (TAS), total oxidant status (TOS), and the oxidative stress index (OSI). MDA (Cat. No. YLA0029RA), SOD (Cat. No. YLA0115RA), CAT (Cat. No. YLA0123RA), GSH (Cat. No. YLA0121RA), GSH-Px (Cat. No. YLA0119RA), and PC (Cat. No. YLA0002RA) were assayed using rat-specific ELISA kits (YL-Biont, Shanghai YL Biotech Co., Ltd., Shanghai, China). TAS (Elabscience, Wuhan, China; Cat. No. E-BC-K801-M) and TOS (Elabscience; Cat. No. E-BC-K802-M) were determined by an automated colorimetric method, and OSI was calculated as the TOS/TAS ratio and expressed in arbitrary units. SOD, CAT and GSH-Px were quantified as protein concentrations by immunoassay rather than as catalytic activities.

### 4.5. Western Blot Analysis

Total protein was extracted from renal tissue by homogenization in ice-cold radioimmunoprecipitation assay (RIPA) buffer containing protease and phosphatase inhibitors, and protein concentration was determined by the BCA (Bio-Rad, Hercules, CA, USA) assay. Equal protein loads (50 µg per lane) were resolved on 10–12% sodium dodecyl sulfate–polyacrylamide gels and transferred to PVDF membranes [38,39]. Membranes were blocked in 5% non-fat dry milk and probed with primary antibodies against Bax (AF0120, Affinity Biosciences, Cincinnati, OH, USA; 1:500), Bcl-2 (AF6139, Affinity Biosciences; 1:500), p62/SQSTM1 (AF5384, Affinity Biosciences; 1:500), V-cam (DF6082, Affinity Biosciences; 1:500), and β-actin (sc-47778, Santa Cruz Biotechnology, Dallas, TX, USA; 1:1000). After incubation with horseradish peroxidase-conjugated secondary antibodies (410406, BioLegend, San Diego, CA, USA; 1:1000), bands were visualized by chemiluminescence on an imaging system, quantified in ImageJ (ImageJ2x 2.1.4.7) and normalized to β-actin [40,41]. For each group, renal tissue homogenates from five animals were pooled into a single composite sample, which was blotted in five replicate runs (*n* = 5 technical replicates per group); this design is a semi-quantitative, confirmatory analysis and does not capture inter-animal biological variability (see Section 4.8 and Discussion for this limitation). Representative immunoblots are shown in Figure 3.

### 4.6. Histopathological Examination

Renal tissue was fixed in 10% neutral-buffered formalin, processed routinely, embedded in paraffin, and sectioned at 4 µm. Sections were stained with hematoxylin and eosin (H&E) and examined by light microscopy. Glomerular congestion, tubular epithelial desquamation, hydropic degeneration, and medullary congestion were graded semiquantitatively according to severity, and the resulting scores used for statistical comparison [42,43]. Glomerular tuft diameter and Bowman’s space width were measured histomorphometrically in 20 randomly selected cortical glomeruli per section using a digital image analysis system [44]. All histopathological and histomorphometric evaluation was performed by experienced histologists blinded to group allocation.

### 4.7. Immunohistochemical Analysis

Apoptotic activity was assessed by caspase-3 immunohistochemistry. Paraffin sections (4 µm) were deparaffinized, rehydrated, and subjected to antigen retrieval, and endogenous peroxidase activity quenched with hydrogen peroxide. Sections were incubated with anti-caspase-3 primary antibody (CASP3 Polyclonal Antibody, E-AB-13815, Elabscience Biotechnology Inc., Houston, TX, USA; 1:200), visualized using an HRP-based detection system with 3,3′-diaminobenzidine as chromogen, counterstained with Mayer’s hematoxylin, and examined by light microscopy. Immunoreactivity was scored semiquantitatively by the H-score method, which combines the proportion of positively stained cells with staining intensity [45].

### 4.8. Statistical Analysis

Analyses were performed in GraphPad Prism 10.0 (GraphPad Software, Boston, MA, USA). Normality was assessed by the Shapiro–Wilk test and homogeneity of variance by the Brown–Forsythe test (the median-centered form of Levene’s test). Normally distributed data are given as mean ± standard deviation (SD) and non-normally distributed data as median with interquartile range (IQR).

Three-group comparisons used one-way ANOVA for normally distributed variables and the Kruskal–Wallis test otherwise. Where the overall comparison reached significance, Tukey’s multiple-comparisons test was applied to parametric data and Dunn’s test to non-parametric data. Western blot data, which derive from technical replicates of a single pooled sample per group, were analyzed in the same way but are reported descriptively, as they do not capture inter-animal variability (see Section 4.5). Mortality was compared across the three groups by the Fisher–Freeman–Halton exact test. Baseline and final body weights were analyzed by one-way ANOVA, and kidney weight by Kruskal–Wallis because it failed the normality assumption. Body weight change (final minus baseline) and relative kidney weight (kidney weight normalized to final body weight) were normally distributed and compared by one-way ANOVA with Tukey’s post hoc test. All tests were two-sided and *p* < 0.05 was taken as significant. The Fisher–Freeman–Halton exact test was computed with the assistance of a generative artificial intelligence tool (see Acknowledgments); all other analyses were performed in GraphPad Prism.

## 5. Conclusions

VOR produced partial renoprotection against experimental renal ischemia–reperfusion injury at biochemical, molecular, and histopathological levels. The effect was associated chiefly with reduced lipid peroxidation, suppression of V-cam, a shift of the Bax/Bcl-2 balance toward cell survival, restoration of p62/SQSTM1, and decreased caspase-3 immunoreactivity, although the immunoblot findings derive from pooled samples and are semi-quantitative. Because BUN was not significantly reduced, IL-6 did not respond as hypothesized, and serum CR, TNF-α, IL-1β, and most antioxidant parameters were unaffected—and because mortality and weight loss were numerically greatest in the treated group—these findings should be read as preliminary and pathway-selective rather than as evidence of broad protection. Further work incorporating multiple dose regimens, a drug-only control arm, extended reperfusion, formal tolerability assessment, and fuller characterization of autophagy and apoptosis will be required to establish mechanism and to judge translational potential.

## Figures and Tables

**Figure 1:**
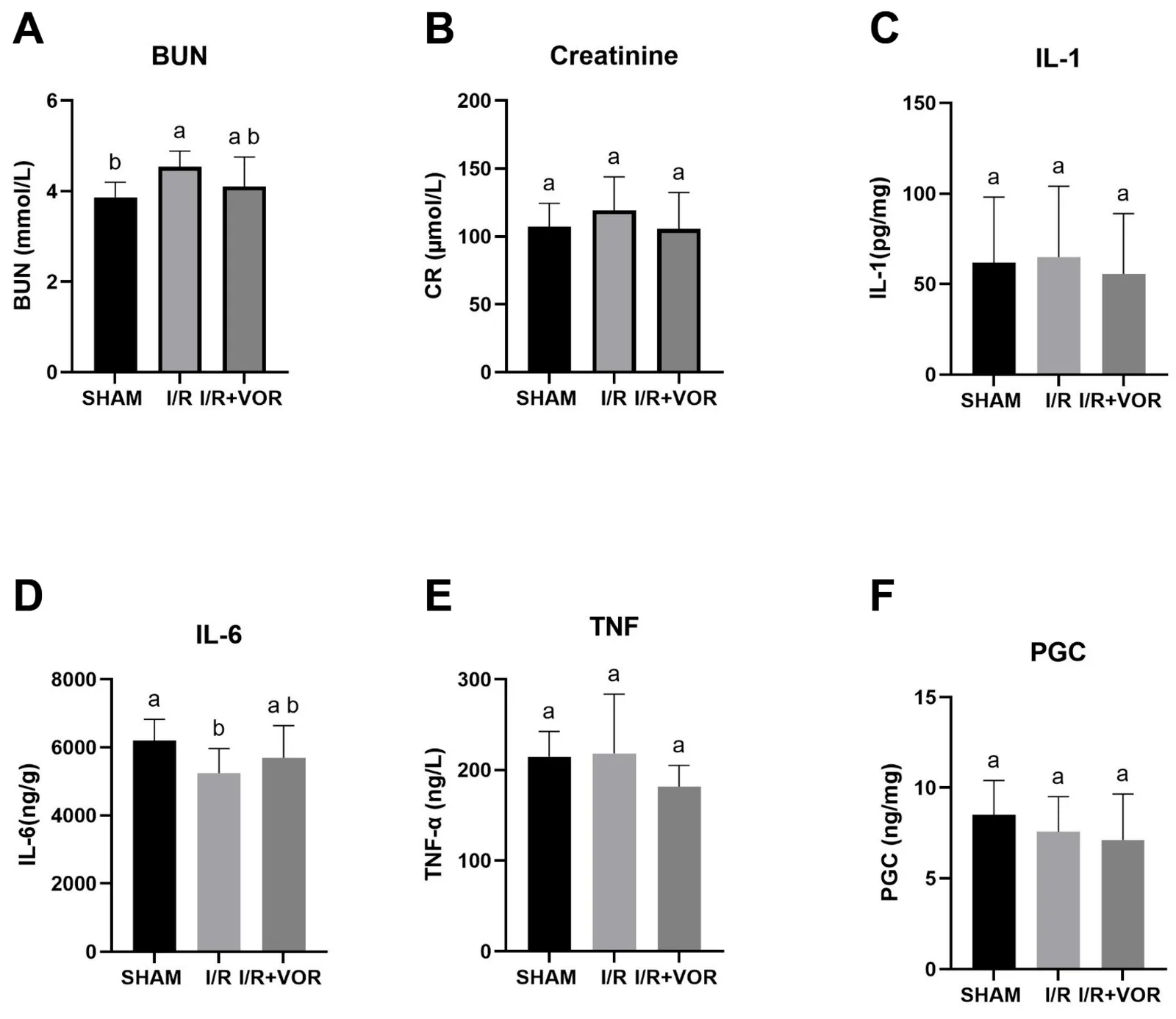
Effects of VOR on biochemical and inflammatory markers in renal ischemia–reperfusion injury: (**A**) blood urea nitrogen, BUN (mmol/L); (**B**) creatinine, CR (µmol/L); (**C**) interleukin-1β, IL-1β (pg/mg tissue); (**D**) interleukin-6, IL-6 (ng/g tissue); (**E**) tumor necrosis factor-α, TNF-α (ng/L); (**F**) peroxisome proliferator-activated receptor gamma coactivator-1 alpha, PGC-1α (ng/mg tissue). Serum urea data were excluded from this figure and from the analysis (see Section 4.4 for details). Data are mean ± SD. Different letters above bars (a, b) denote significant differences between groups (*p* < 0.05); groups sharing a letter do not differ. Comparisons used one-way ANOVA followed by Tukey’s multiple-comparisons test. Sham (*n* = 11); I/R (*n* = 11); VOR+I/R (*n* = 9).

**Figure 2:**
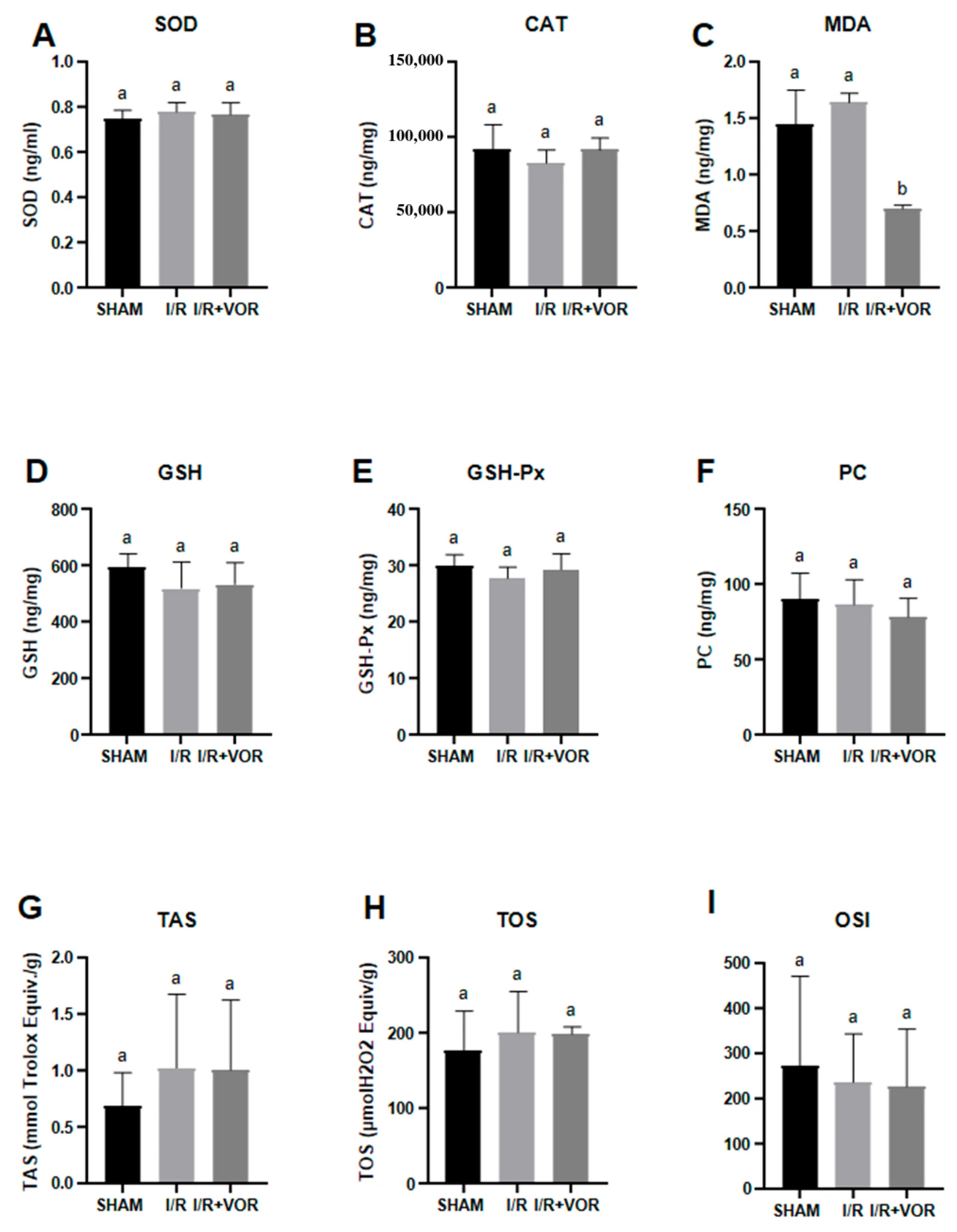
Effects of VOR on oxidative stress parameters following renal ischemia–reperfusion injury: (**A**) superoxide dismutase, SOD (ng/mL); (**B**) catalase, CAT (ng/mg tissue); (**C**) malondialdehyde, MDA (ng/mg tissue); (**D**) reduced glutathione, GSH (ng/mg tissue); (**E**) glutathione peroxidase, GSH-Px (ng/mg tissue); (**F**) protein carbonyl, PC (ng/mg tissue); (**G**) total antioxidant status, TAS (mmol Trolox equivalent/g tissue); (**H**) total oxidant status, TOS (µmol H_2_O_2_ equivalent/g tissue); (**I**) oxidative stress index, OSI (arbitrary units). Data are mean ± SD. Different letters above bars (a, b) denote significant differences between groups (*p* < 0.05); groups sharing a letter do not differ. Comparisons used one-way ANOVA followed by Tukey’s multiple-comparisons test. Sham (*n* = 11); I/R (*n* = 11); VOR+I/R (*n* = 9).

**Figure 3:**
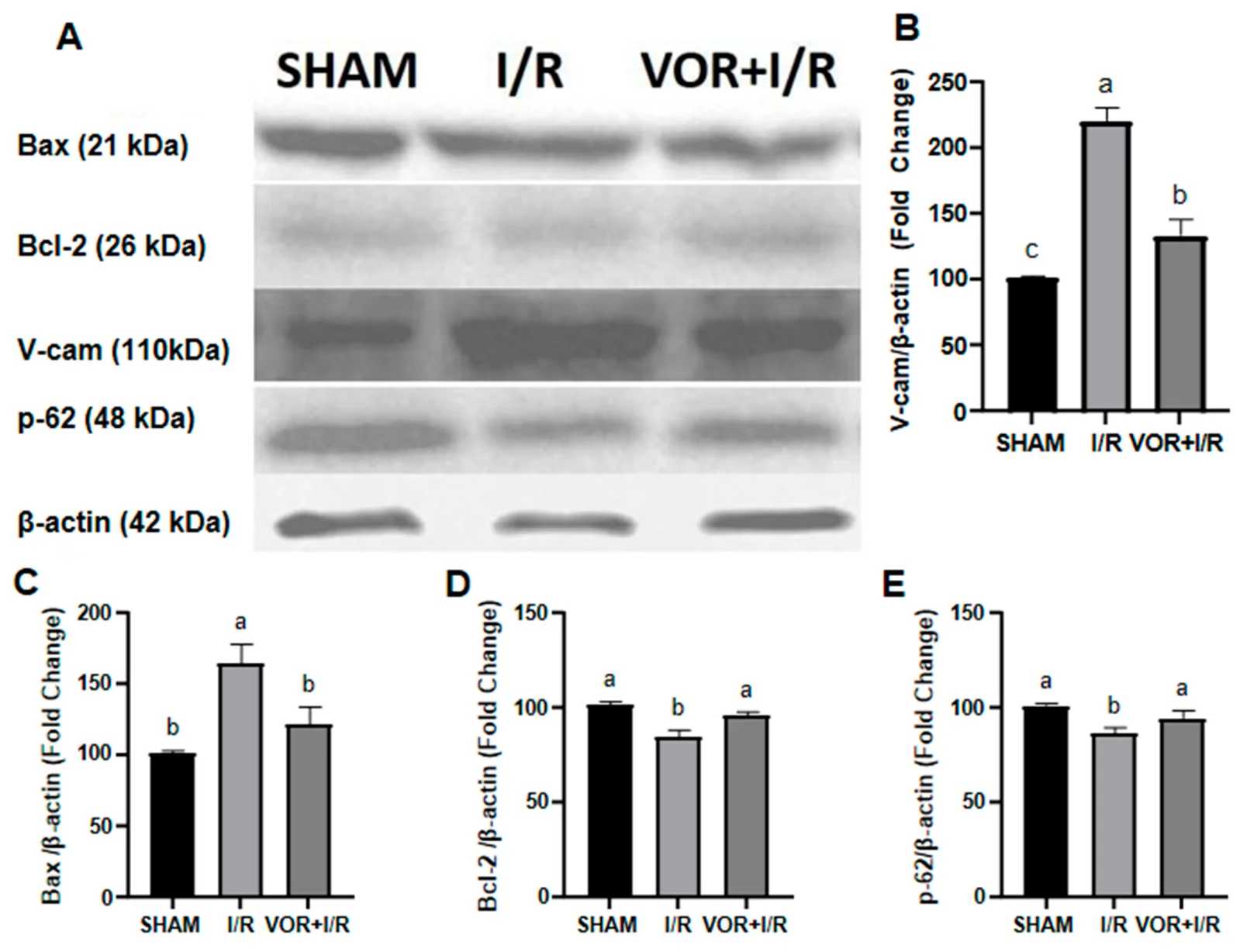
Effects of VOR on V-cam, Bax, Bcl-2, and p62 protein expression in renal ischemia–reperfusion injury: (**A**) representative immunoblots for Bax, Bcl-2, V-cam, p62/SQSTM1, and β-actin; (**B**) V-cam/β-actin; (**C**) Bax/β-actin; (**D**) Bcl-2/β-actin; (**E**) p62/β-actin. Band intensities were normalized to β-actin and are expressed as fold change over the Sham group (mean ± SD). Each lane represents a pooled homogenate from five animals per group, blotted in five replicate runs (*n* = 5 technical replicates per group; see Section 4.5 and Section 4.8). Different letters above bars (a, b, c) denote differences between pooled samples across technical replicates (*p* < 0.05); groups sharing a letter do not differ. These letters describe assay-level differences and do not represent inter-animal statistical significance. Comparisons used one-way ANOVA followed by Tukey’s multiple-comparisons test.

**Figure 4:**
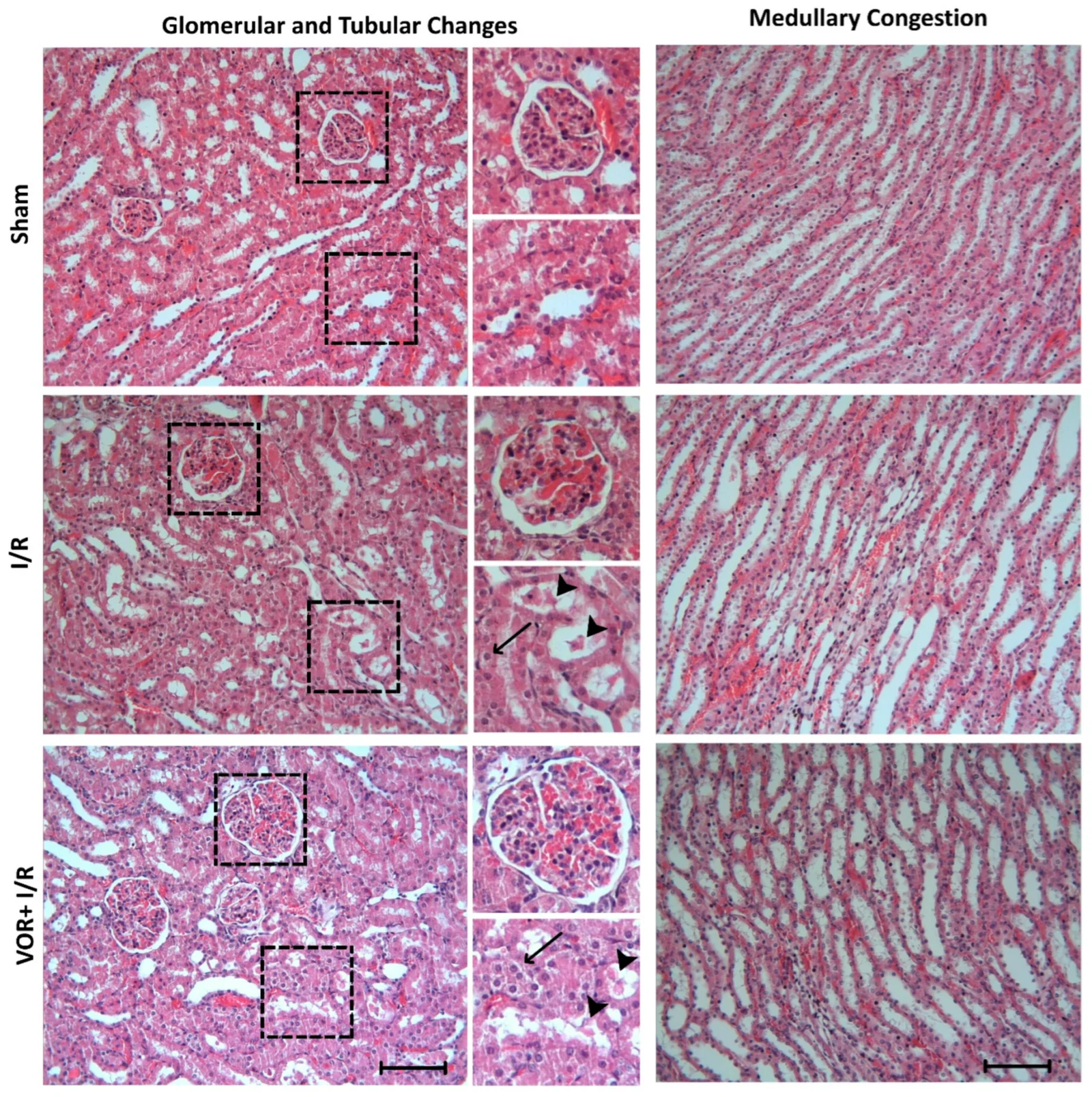
Representative histopathology of renal tissue after ischemia–reperfusion injury and VOR treatment. Renal architecture was largely preserved in the Sham group, with only mild tubular epithelial desquamation and minimal glomerular change. The I/R group showed prominent tubular epithelial desquamation, hydropic degeneration of tubular epithelial cells, epithelial debris within tubular lumina, and congested glomeruli with erythrocyte-filled capillaries. In the VOR+I/R group, tubular epithelial desquamation, cellular swelling, and glomerular congestion were attenuated relative to I/R, though still detectable. Medullary congestion was present in all groups but less pronounced in Sham and VOR+I/R than in I/R, without reaching statistical significance. Dashed boxes indicate the regions shown enlarged in the adjacent images (upper: glomerulus; lower: renal tubules). Arrowheads indicate epithelial debris; arrows indicate hydropic degeneration. H&E; original magnification ×200; scale bar = 100 µm.

**Figure 5:**
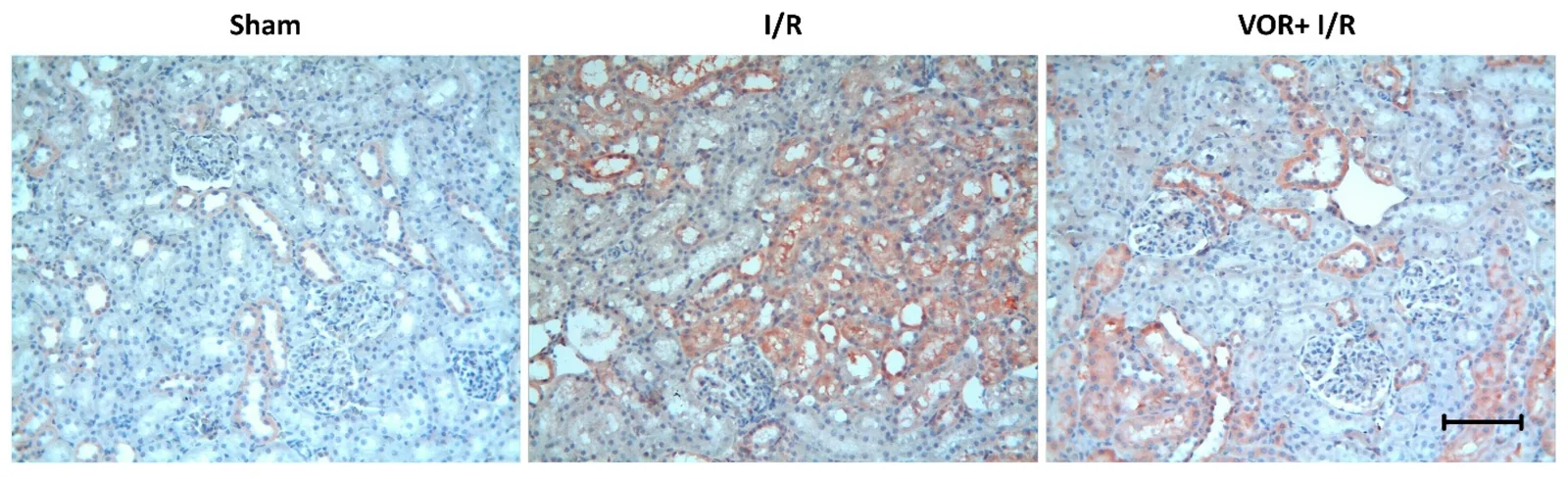
Representative caspase-3 immunohistochemistry in renal tissue. Caspase-3 positivity was mild in the Sham group and markedly increased in the I/R group. VOR treatment substantially reduced immunoreactivity relative to I/R. Caspase-3 immunostaining; original magnification ×200; scale bar = 100 µm. Immunoreactivity was quantified semiquantitatively by H-score and compared between groups by Kruskal–Wallis followed by Dunn’s multiple-comparisons test; quantitative values appear in Table 3. Sham (*n* = 11); I/R (*n* = 11); VOR+I/R (*n* = 9).

**Table 1:** Animal allocation, mortality, and final sample size.

Group	Initial (*n*)	Deaths (*n*)	Analyzed (*n*)	Mortality (%)
Sham	12	1	11	8.3
I/R	12	1	11	8.3
VOR+I/R	12	3	9	25.0
Total	36	5	31	13.9

Mortality (%) was calculated as deaths divided by the initial number of animals per group. Sham, right nephrectomy without renal ischemia; I/R, ischemia–reperfusion; VOR+I/R, vortioxetine-treated ischemia–reperfusion.

**Table 2:** Body and kidney weight by experimental group.

Parameter	Sham (*n* = 11)	I/R (*n* = 11)	VOR+I/R (*n* = 9)	Test; *p* Value
Baseline body weight (g) ^a^	220.7 ± 18.9	225.4 ± 15.5	220.8 ± 16.4	ANOVA; *p* = 0.773
Final body weight (g) ^a^	214.8 ± 17.8	224.0 ± 15.2	209.9 ± 18.5	ANOVA; *p* = 0.188
Kidney weight (g) ^b^	0.879 (0.808–0.918)	0.870 (0.834–0.897)	0.803 (0.732–0.914)	Kruskal–Wallis; *p* = 0.697
Body weight change (g) ^c^	−5.9 ± 3.6	−1.4 ± 3.3	−10.9 ± 8.1	ANOVA; *p* = 0.002
Relative kidney weight (mg/g) ^d^	4.04 ± 0.34	3.81 ± 0.41	3.93 ± 0.55	ANOVA; *p* = 0.471

^a^ Mean ± SD; normality confirmed by Shapiro–Wilk in all groups (*p* > 0.05); compared by one-way ANOVA. ^b^ Kidney weight in the I/R group failed the normality assumption (Shapiro–Wilk *p* = 0.031), so data are median (IQR) and were compared by Kruskal–Wallis. ^c^ Final minus baseline body weight per animal; normally distributed (Shapiro–Wilk *p* > 0.05) with homogeneous variances (Brown–Forsythe *p* = 0.143); Tukey’s post hoc test showed a difference between I/R and VOR+I/R (*p* = 0.001), with Sham not differing from either (*p* > 0.05). ^d^ Kidney weight (mg) divided by final body weight (g). Kidney weight refers to the remaining left (ischemic) kidney after right nephrectomy. Homogeneity of variance was confirmed by Brown–Forsythe for all variables in this table (*p* > 0.05). All tests were two-sided with significance at *p* < 0.05.

**Table 3:** Histomorphometric, histopathological, and immunohistochemical findings by experimental group.

Parameter	Sham (*n* = 11)	I/R (*n* = 11)	VOR+I/R (*n* = 9)	*p* Value
Bowman’s space width (µm)	11.90 (9.20–15.30)	15.60 (11.20–20.50) ^a^	11.50 (9.30–13.80)	<0.05
Glomerular tuft diameter (µm)	83.00 (73.10–88.80)	83.90 (79.80–87.50)	86.30 (80.40–95.00)	>0.05
Glomerular congestion score	0.47 (0.35–0.50)	1.37 (0.85–1.75) ^a^	0.95 (0.35–2.00)	<0.05
Tubular desquamation score	0.65 (0.20–0.70)	1.85 (1.20–2.60) ^a^	1.20 (0.80–2.00) ^b^	<0.05
Hydropic degeneration score	0.10 (0.00–0.20)	1.70 (1.10–2.60) ^a^	1.35 (1.20–1.90)	<0.05
Caspase-3 immunoreactivity (H-score)	1.45 (0.50–2.40)	5.65 (3.90–8.80) ^a^	2.95 (1.50–4.80) ^b^	<0.05

Data are median (IQR). Congestion, desquamation, and hydropic degeneration were graded semiquantitatively on H&E-stained sections; Bowman’s space width and glomerular tuft diameter were measured in 20 randomly selected glomeruli per section; caspase-3 immunoreactivity was assessed by H-score. None of these variables met the normality assumption, so groups were compared by Kruskal–Wallis followed by Dunn’s multiple-comparisons test. ^a^ *p* < 0.05 versus Sham; ^b^ *p* < 0.05 versus I/R. Medullary congestion scores did not differ between groups (*p* > 0.05).

## Data Availability

The data presented in this study are available on request from the corresponding author. The data are not publicly available because they form part of an ongoing institutional research project.

## Supplementary Materials

The following supporting information can be downloaded at https://www.mdpi.com/article/10.3390/ijms27198679/s1.
